# Research on Inertial Navigation and Environmental Correction Indoor Ultra-Wideband Ranging and Positioning Methods

**DOI:** 10.3390/s24010261

**Published:** 2024-01-02

**Authors:** Chunhua Han, Shunbiao Xue, Li Long, Xiongquan Xiao

**Affiliations:** Faculty of Transportation Engineering, Kunming University of Science and Technology, Kunming 650500, China; hanunhua@kmust.edu.cn (C.H.); 20212206034@stu.kust.edu.cn (L.L.); 20222206085@stu.kust.edu.cn (X.X.)

**Keywords:** ultra-wideband, inertial navigation, digital environment, high-precision positioning

## Abstract

In contrast to outdoor environments, indoor positioning encounters signal propagation disruptions due to the presence of buildings, resulting in reduced accuracy and, at times, the inability to determine a location accurately. This research, leveraging the robust penetrative capabilities of Ultra-Wideband (UWB) signals in non-line-of-sight (NLOS) scenarios, introduces a methodology for refining ranging outcomes through a combination of inertial navigation and environmental adjustments to achieve high-precision spatial positioning. This approach systematically enhances the correction of signal propagation errors through walls. Initially, it digitalizes the spatial setting, preserving the error correction parameters. Subsequently, it employs inertial navigation to estimate spatial coordinates and delineate signal propagation pathways to achieve precise ranging results. It iteratively hones the positioning outcomes for enhanced precision. Empirical findings demonstrate that within NLOS conditions, compared to standalone UWB positioning and IMU/UWB fusion positioning using the ESKF algorithm, this positioning technique significantly enhances planar positioning accuracy while achieving a marginal elevation accuracy improvement, albeit with some residual deviations from actual values. Furthermore, this positioning methodology effectively rectifies results in NOLS settings, paving the way for a novel approach to optimize indoor positioning through UWB technology.

## 1. Introduction

With the introduction and promotion of concepts such as the digital economy and digital twins, the potential of data elements is activated, driving changes in production methods, lifestyles, and governance through digital transformation. Spatial positioning data is an indispensable element for digitally describing the real world. In outdoor environments, the combined navigation system composed of the Global Satellite Navigation System (GNSS) and Inertial Navigation System (INS) can achieve positioning accuracy up to decimeters or even centimeters [1]. However, in indoor environments, due to the absence of GNSS signals, positioning methods relying on GNSS and its related technologies can no longer provide high-precision positioning services [2].

With the maturity of communication technology, technologies such as Ultra-Wideband (UWB) [3,4], WiFi [5,6], Bluetooth Low Energy (BLE) [7,8], Radio Frequency Identification (RFID) [9,10,11], and ultrasound [12] have been able to achieve indoor positioning and have been widely used in various fields such as emergency management [13], smart energy management [14], smart HVAC control [15], point-of-interest identification [16], occupancy prediction [17], and equipment management [18]. However, these indoor positioning systems each have their own characteristics, and considering factors such as positioning accuracy, reliability, and installation cost, different positioning systems have their own shortcomings. For example, the accuracy of WiFi positioning is at the meter level. It can utilize existing infrastructure without additional hardware investment, but the positioning accuracy is affected by factors such as the number of access points and signal strength [6]. In addition, the accuracy of BLE positioning is at the meter level, and its advantage lies in low cost, but it has a slow transmission speed and a short propagation distance [8]. The accuracy of RFID positioning is also at the meter level, but it is seriously affected by multipath, resulting in a short positioning distance [11]. In addition, the accuracy of ultrasound positioning is at the decimeter level. It is affected by the propagation characteristics of sound waves and encounters obstacles and multipath interference may cause a decrease in accuracy [12]. Finally, the accuracy of UWB positioning can reach the centimeter level. It uses pulses with a width of only nanoseconds as communication signals, has a very high time resolution, and also has certain anti-multipath capabilities and penetration [4]. Therefore, it can achieve high-precision positioning results in complex indoor environments.

In line-of-sight (LOS) environments, UWB can achieve ranging accuracy up to the centimeter level [3]. However, in complex indoor environments, there are obstacles, and the UWB signal propagation process will involve refraction, reflection, and different propagation rates in different media, leading to non-line of sight (NLOS) errors that significantly affect its distance measurement accuracy, resulting in a decrease in the position coordinate precision obtained through the calculation of distance measurement results based on base station coordinates and the distance between base station and tag. To address the challenges posed by NLOS interference in positioning methods based on UWB, several effective solutions have been proposed. These include using Kalman filtering, unscented Kalman filtering, and particle filtering to refine raw ranging data and minimize NLOS error influences [19,20,21,22]. Furthermore, leveraging both the Time of Arrival (TOA) and Angle of Arrival (AOA) for mixed ranging has shown efficacy in mitigating the impact of NLOS errors [23,24]. Another effective method involves utilizing path loss models to compensate for the raw ranging data, which reduces the implications of NLOS errors [25,26,27]. Additionally, the integration of UWB and the Inertial Navigation System (INS) offers a dual advantage. It not only harnesses the INS computations to diminish NLOS error effects in UWB but also exploits UWB’s ranging or computational data to counteract the swift error accumulation in INS. This synergy results in heightened navigation positioning precision and a richer set of navigation data [28,29]. However, ordinary inertial navigation sensors have significant measurement noise, and positioning errors gradually increase over time or distance, resulting in deviations from the actual position. Therefore, increasing the range measurement accuracy of UWB can improve the positioning accuracy of UWB/INS integration in NLOS environments.

In NLOS environments, UWB ranging inaccuracies predominantly stem from two factors: geometric distance errors, which result from the extra delay as pulse signals traverse walls, and peak shift errors caused by distortions in the pulse waveform. The geometric distance error is mainly determined by the propagation distance of the signal within wall obstructions and the material. The distance between the transmitter and receiver nodes has a minimal effect on this error. On the other hand, waveform distortions are primarily influenced by the wall’s relative permittivity and heterogeneity [30]. Literature [27] analyzed the distance errors of UWB signals passing through walls in a complete NLOS environment and integrated the path loss model and Kalman distance filtering model into the positioning algorithm, yielding significant improvements in positioning results. However, this method distinguishes the environment using a threshold, capable of differentiating only between LOS and NLOS scenarios, and cannot provide precise ranging compensation in situations with multiple obstructions.

In summary, based on the existing UWB/INS combined positioning and UWB signal propagation characteristics, this study incorporates the digital environment of the positioning scenario into the system, providing correction parameters for the ranging errors caused by UWB signal propagation through walls, thereby precisely compensating for NLOS errors in complex indoor environments. The method improves the errors in signal propagation through walls by first digitizing the positioning environment and recording the error correction parameters. Subsequently, inertial navigation is used to estimate the spatial position, infer the signal propagation path, and precisely correct the ranging results. Finally, by repeatedly iterating and correcting the positioning results, higher accuracy is achieved. Empirical experiment results indicate that compared to standalone UWB positioning and IMU/UWB fusion positioning using the ESKF algorithm, this positioning method significantly improves planar positioning accuracy in NLOS environments and slightly enhances altitude accuracy, although significant discrepancies still remain. This positioning method effectively corrects the positioning results in NOLS environments and offers a new optimization approach for indoor positioning based on UWB. High-precision indoor positioning contributes to providing high-quality indoor navigation data, enabling tracking and supervision of personnel and equipment, thereby enhancing emergency response capabilities and the level of security assurance.

## 2. Methods

### 2.1. Data Collection

#### 2.1.1. Digital Environment and Construction Method

The digital environment is a numerical description of building components in a positioning environment. It focuses on describing the position and geometric information of building components in the navigation coordinate system, as well as the correction information of ranging results when the ranging signal penetrates the structure. During positioning, the penetration of components by ranging signals can be deduced by estimating the signal propagation path, obtaining corresponding correction information and making the ranging results closer to the true values. This provides precise positioning support for the positioning system, demonstrating superior performance in complex indoor environments.

**Figure 1:**
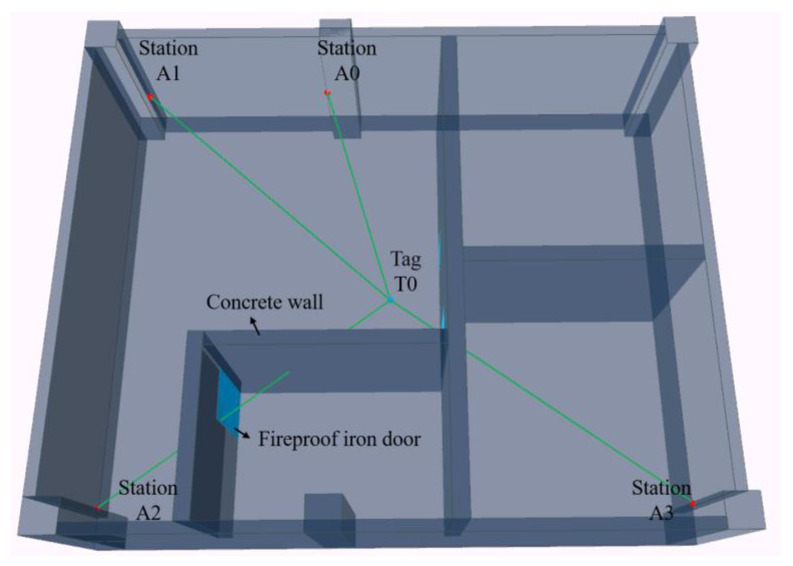
The visualization representation of digital environments.

When constructing the digital environment, first, collect building component data and establish a three-dimensional digital model of components based on the Boundary Representation model (B-Rep). This process ensures the consistency of the digital model with the actual building. Secondly, mark relevant attribute information of the building in the model, including basic properties of construction materials, ranging signal propagation properties, and correction information of ranging results. Figure 1 visualizes the digital environment. Through spatial calculations involving estimated paths and component digital models, it is discernible that tag T0 is in LOS conditions with base stations A0 and A1, while in NLOS conditions with base stations A2 and A3, the ranging signal between T0 and A2 is interfered by an iron door and concrete wall, and the signal between T0 and A3 is interfered by an iron door.

#### 2.1.2. Ranging Information Collection

**Figure 2:**
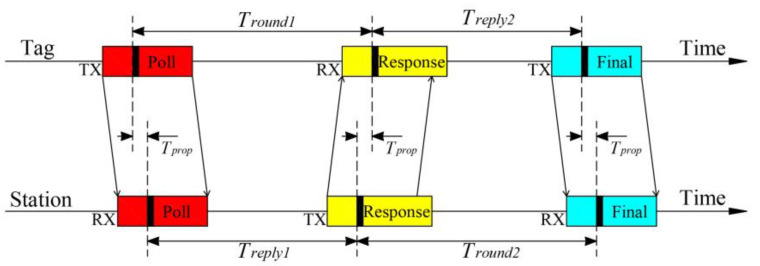
The schematic diagram illustrating the principle of distance measurement by ADS-TWR.

This paper employs Asymmetric Double-Sided Two-Way Ranging (ADS-TWR) [31] based on Time of Flight (TOF) for distance measurement, as illustrated in Figure 2. Therefore, the formula for calculating signal flight time is given by Equation (1), and the formula for measuring distance is given by Equation (2), where c represents the signal propagation speed in the air with a value of 299,792,458 m/s. This method calculates the signal transmission time by measuring the time difference between two round trips, eliminating the need for clock synchronization between base stations and tags or between base stations, leading to simple equipment deployment. Additionally, this ranging method can effectively mitigate the influence of crystal oscillator drift without requiring identical delays at both ends of the transmitter and receiver.

(1) $${\hat{T}}_{prop}=\frac{T_{round1}\times T_{round2}-T_{reply1}\times T_{reply2}}{T_{round1}+T_{round2}+T_{reply1}+T_{reply2}}$$

(2) $$L={\hat{T}}_{prop}\times c$$

#### 2.1.3. Sports Information Collection

In this paper, real-time collection of acceleration and angular velocity is conducted using an Inertial Measurement Unit (IMU) composed of a three-axis accelerometer and gyroscope to obtain the device’s motion information in three-dimensional space. The accelerometer outputs signals for three-axis acceleration, while the gyroscope outputs signals for three-axis angular velocity. After processing these signals, the object’s pose and position can be inferred.

### 2.2. Data Processing Methods

#### 2.2.1. Ranging Data Preprocessing

**Figure 3:**
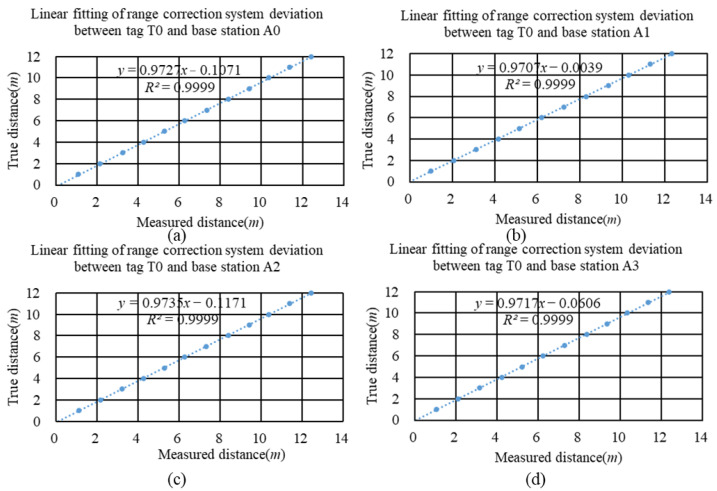
WUB Ranging System Bias Correction. (**a**) Linear fitting of range correction system deviation between tag T0 and base station A0 in LOS environment. (**b**) Linear fitting of range correction system deviation between tag T0 and base station A1 in LOS environment. (**c**) Linear fitting of range correction system deviation between tag T0 and base station A2 in LOS environment. (**d**) Linear fitting of range correction system deviation between tag T0 and base station A3 in LOS environment.

In indoor scenes, the propagation of UWB signals is influenced by LOS and NLOS environments. In LOS environments, UWB signals can be considered to propagate almost linearly with minimal or no multipath propagation. The signal propagation is reliable, resulting in high-ranging accuracy, and the research on ranging error in positioning systems has obtained a relatively uniform Gaussian distribution model [32,33]. However, due to the system deviation between UWB distance measurement results and actual values caused by different devices in different usage environments, devices should be calibrated under the LOS environment before building the positioning system to obtain high-precision distance measurements. The calibration method was as follows: Data were collected 500 times at every 1 m interval within a range from 1 m to 12 m. Then, the mode of the collected results was used as the measured distance. This measured distance was linearly fitted against the true distance, with the fitting results shown in Figure 3.

#### 2.2.2. Distance Filtering Method

ADS-TWR ranging is based on the calculation of the round-trip time of pulse signals between the base station and the mobile tag, and the measurement results contain noise. In addition, environmental corrections resulting from estimated position errors can also cause ranging results to deviate from the true values. Therefore, Kalman filtering is employed to smooth the ranging results.

In this paper, the distance between the mobile tag and the base station, along with the rate of change in distance, is taken as the state parameters $x_{k}={\left[\begin{array}{cc} d_{k} & v_{k} \end{array}\right]}^{T}$; the state equation is as follows:

(3) $${\hat{x}}_{k}^{-}=F{\hat{x}}_{k-1}+\omega_{k-1}$$

where $F=\left[\begin{array}{cc} 1 & \Delta T_{k}^{uwb}\\ 0 & 1 \end{array}\right]$ is the state matrix. $\Delta T_{k}^{uwb}$ is the UWB data sampling interval. $\omega_{k-1}$ represents the process noise at time k−1, and its covariance matrix is *Q* = 0.018.

Taking the UWB ranging value at time k as the observation $z_{k}$, the measurement equation is:

(4) $$z_{k}=H_{k}x_{k}+u_{k}$$

where $z_{k}=d_{k}^{uwb}$ is the ADS-TWR ranging value at time k. $H=\left[\begin{array}{cc} 1 & 0 \end{array}\right]$ is the measurement matrix. $u_{k}$ is the process noise at time k−1, and its covariance matrix is *R* = 0.542.

Based on the state and measurement equations, state and measurement updates are performed through Kalman filtering, with the computation formula as follows:

(5) $$\left.\begin{array}{l} P_{k}^{-}=FP_{k-1}A^{T}+Q\\ K_{k}=P_{k}^{-}H_{k}^{T}{\left(H_{k}P_{k}^{-}H_{k}^{T}+R\right)}^{-1}\\ {\hat{x}}_{k}={\hat{x}}_{k}^{-}+K_{k}\left(z_{k}-H_{k}{\hat{x}}_{k}^{-}\right)\\ P_{k}=\left(I-K_{k}H_{k}\right)P_{k}^{-} \end{array}\right\}$$

#### 2.2.3. Inertial Navigation Data Processing

The output values from the gyroscope and accelerometer in the inertial navigation system are given in the body coordinate system (B-coordinate system) and must be converted to the navigation coordinate system (N-coordinate system) to be utilized for correcting fused positioning data. The navigation coordinate system is defined as the East-North-Up (ENU) coordinate system, and within this framework, the heading, pitch, and roll angles of the body coordinate system are designated as φ/θ/γ, respectively. The transformation matrix $C_{B}^{N}$ for converting from the body coordinate system to the navigation coordinate system is defined in Equation (6). It is important to note that the multiplication of matrices is not interchangeable because each product represents a different order of rotation. Attitude updating is performed by real-time computation of the transformation matrix based on IMU output values, and this paper utilizes the Mahony complementary filter algorithm [34] to solve for φ/θ/γ.

(6) $$C_{B}^{N}=\left[\begin{array}{ccc} 1 & 0 & 0\\ 0 & \cos\gamma & \sin\gamma \\ 0 & -\sin\gamma & \cos\gamma \end{array}\right]\left[\begin{array}{ccc} \cos\theta & 0 & -\sin\theta \\ 0 & 1 & 0\\ \sin\theta & 0 & \cos\theta \end{array}\right]\left[\begin{array}{ccc} \cos\varphi & \sin\varphi & 0\\ -\sin\varphi & \cos\varphi & 0\\ 0 & 0 & 1 \end{array}\right]$$

The accelerometer outputs a value $a^{B}=\left[\begin{array}{ccc} a_{x}^{B} & a_{y}^{B} & a_{z}^{B} \end{array}\right]$ in the body coordinate system; thus, the formula for calculating the three-axis acceleration in the navigation coordinate system is given by Equation (7), where g is the acceleration due to gravity.

(7) $$a^{N}=C_{B}^{N}a^{B}-{\left[\begin{array}{ccc} 0 & 0 & g \end{array}\right]}^{T}$$

Integrating the acceleration in the navigation coordinate system yields the real-time velocity $v^{N}\left(t\right)$ of the carrier, and integrating the velocity gives the position $x^{N}\left(t\right)$.

(8) $$v^{N}\left(t\right)=\left[\begin{array}{c} v_{x}^{N}\left(t-1\right)\\ v_{y}^{N}\left(t-1\right)\\ v_{z}^{N}\left(t-1\right) \end{array}\right]+\frac{1}{2}\left(\left[\begin{array}{c} a_{x}^{N}\left(t-1\right)\\ a_{y}^{N}\left(t-1\right)\\ a_{z}^{N}\left(t-1\right) \end{array}\right]+\left[\begin{array}{c} a_{x}^{N}\left(t\right)\\ a_{y}^{N}\left(t\right)\\ a_{z}^{N}\left(t\right) \end{array}\right]\right)\Delta t$$

(9) $$s^{N}\left(t\right)=\left[\begin{array}{c} s_{x}^{N}\left(t-1\right)\\ s_{y}^{N}\left(t-1\right)\\ s_{z}^{N}\left(t-1\right) \end{array}\right]+\frac{1}{2}\left(\left[\begin{array}{c} v_{x}^{N}\left(t-1\right)\\ v_{y}^{N}\left(t-1\right)\\ v_{z}^{N}\left(t-1\right) \end{array}\right]+\left[\begin{array}{c} v_{x}^{N}\left(t\right)\\ v_{y}^{N}\left(t\right)\\ v_{z}^{N}\left(t\right) \end{array}\right]\right)\Delta t$$

#### 2.2.4. Ranging Result Correction Based on Digital Environment

In NLOS environments, environmental statistical similarity has significantly diminished, and UWB signals undergo multipath propagation effects [35]. During signal propagation, phenomena such as reflection, scattering, and diffraction occur, forming signals at the receiver with multiple paths, resulting in multiple arrival times and different gain signal components, significantly reducing ranging accuracy. This paper fully utilizes the advantages of the digital environment, models and corrects ranging errors corresponding to different types of signal propagation paths to improve ranging accuracy and, consequently, enhance the precision of the positioning system.

A. Method for determining the intersection of the propagation path with building components.

**Figure 4:**
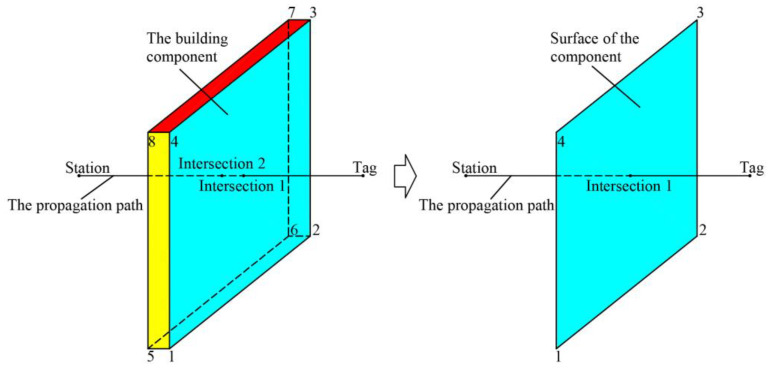
Schematic diagram illustrating the determination of the intersection between the propagation path and building components.

In the digital environment, building components are described as boundary representation models, and the propagation path is approximately depicted as line segments, as shown in Figure 4. Components are represented as three-dimensional geometries composed of six faces, and the propagation path is a three-dimensional line segment from the base station to the tag. In the positioning environment, the positions of the base station and the tag cannot exist inside the components. The problem of the propagation path intersecting with the components is transformed into a problem of the propagation path intersecting with the surface of the components. In other words, when the propagation path intersects with any surface of the components, it is considered that the propagation path intersects with the components. The specific calculation method is as follows: Firstly, calculate the intersection point of the line where the propagation path is located and the plane where the surface of the components is located. If the intersection point does not exist, the propagation path does not intersect with the components. Then, calculate whether the intersection point lies within the topological relationship of the surface of the components. If the intersection point is contained within the surface of the components, then the propagation path intersects with the components; otherwise, the propagation path does not intersect with the components.

B. Ranging Result Correction

**Figure 5:**
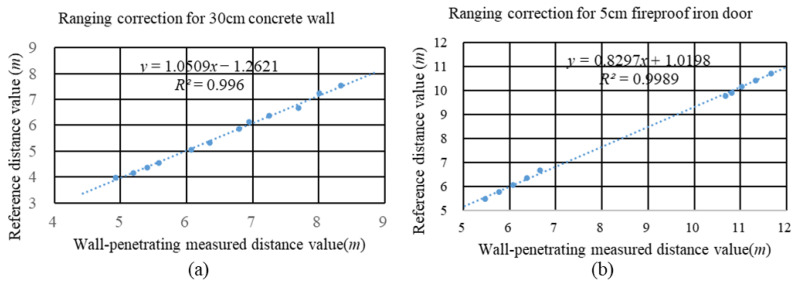
Wall penetration ranging error correction fitting effect chart. (**a**) The distance measurement results of signal penetrating a 30 cm concrete wall were fitted. (**b**) The distance measurement results of signal penetrating a 5 cm fireproof iron door were fitted.

This paper conducts a fitting analysis of ranging data collected at various distances and incidence angles from a 30 cm concrete wall and a 5 cm fireproof iron door in the experimental environment to determine the error correction parameters. According to Figure 5, there is a notable linear correlation between the measured distances through the wall and the reference distances. Hence, by conducting multiple measurements in varied NLOS conditions, we are able to adjust the wall-penetrating distance measurements precisely using a linear fitting approach.

### 2.3. Spatial Position Estimation

**Figure 6:**
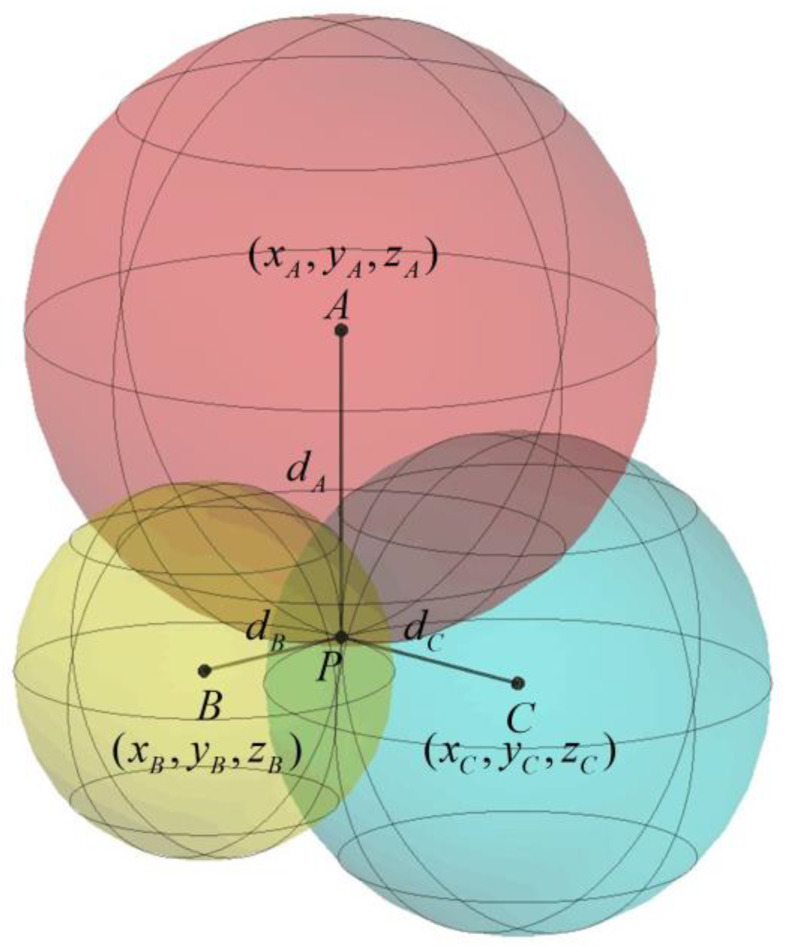
Trilateration Algorithm Schematic.

**Figure 7:**
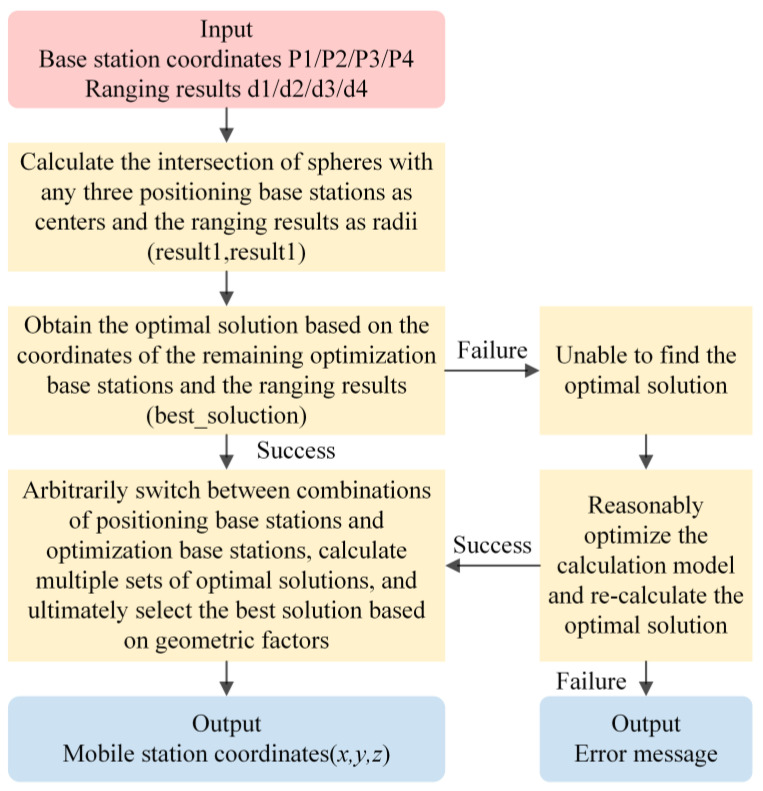
Spatial Position Calculation Algorithm Flowchart.

The spatial position is calculated using a trilateration algorithm [36], the workings of which are depicted in Figure 6. It is assumed that the distances from the reference points $A\left(x_{A},y_{A},z_{A}\right)$, $B\left(x_{B},y_{B},z_{B}\right)$, $C\left(x_{C},y_{C},z_{C}\right)$ to the mobile point $P\left(x,y,z\right)$ are $d_{A}$, $d_{B}$, $d_{C}$ respectively. Spheres with centers at reference points A, B, C, and radii corresponding to these distances should ideally intersect at the mobile point P. However, due to measurement errors in both the reference point coordinates and the UWB range measurements, the spheres may not intersect at a single point. To address this in practice, the number of reference points is increased, and multiple computations are performed to determine the best solution. The method utilized in this paper is the DecaWave-provided algorithm for three-dimensional positioning using four reference points, and its procedure is outlined in Figure 7.

### 2.4. Integrated Positioning Process Design

**Figure 8:**
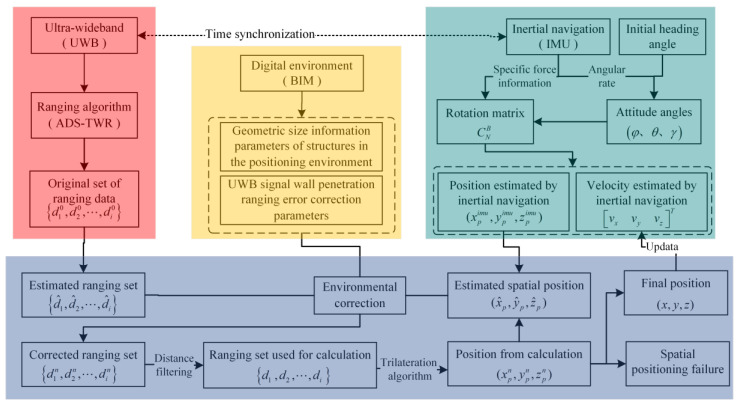
Integrated positioning flowchart.

UWB ranging is affected by NLOS environments, causing a decrease in positioning accuracy in complex indoor environments. Inertial navigation can achieve spatial positioning without relying on external information or emitting energy externally, but its positioning results are only accurate for a short period; over time or increased distance, positioning errors gradually accumulate. Fusion positioning makes full use of data from UWB, IMU, and the digital environment. It is designed with the core concept of tight coupling, primarily relying on UWB arrays. It utilizes IMU to calculate the spatial changes of the tag between consecutive UWB positioning instances. Based on the spatial position derived from IMU, it corrects the ranging errors of UWB in NLOS environments using digital environment information. Finally, it uses the results of two consecutive UWB positioning instances to estimate the motion state of the tag and applies it to the initial state of IMU for spatial change estimation. The positioning process is illustrated in Figure 8. The UWB detection module outputs communication parameters between the tag and each base station, calculating the communication distance. The IMU detection module outputs the tag’s three-axis angular velocity and acceleration, calculating the tag’s orientation, velocity, and estimated position. Firstly, based on the base station coordinates and the estimated position derived from IMU, the pulse signal propagation path is predicted. Then, in the digital environment, spatial bodies intersecting with the propagation path are queried to obtain ranging correction information when the signal penetrates these spatial bodies, and the ranging results are corrected. Next, the corrected ranging results are smoothed using distance filtering methods. Finally, the spatial position of the moving tag is calculated using a trilateration algorithm. Additionally, to reduce the influence of IMU derivation errors on ranging corrections, if the difference between the calculated position and the estimated position exceeds a threshold, the calculated position will be used as the estimated position for a new round of ranging correction and spatial position estimation. The detailed data fusion process is as follows:

(1) Based on the ADS-TWR ranging algorithm, acquire the set of measured distances $\left\{d_{1}^{0},d_{2}^{0},\cdots ,d_{i}^{0}\right\}$ between the mobile tag and each base station tag, which is then taken as the set of ranging estimates $\left\{{\hat{d}}_{1},{\hat{d}}_{2},\cdots ,{\hat{d}}_{i}\right\}$;
(2) Using inertial navigation data to solve for the mobile tag’s attitude angles φ/θ/γ, and derive velocity ${\left[\begin{array}{ccc} v_{x} & v_{y} & v_{z} \end{array}\right]}^{T}$ and spatial position $\left(x_{p}^{imu},y_{p}^{imu},z_{p}^{imu}\right)$, with the position derived from inertial navigation taken as the estimated spatial position $\left({\hat{x}}_{p},{\hat{y}}_{p},{\hat{z}}_{p}\right)$;
(3) Assume that the UWB signal is emitted by the base station tag at $\left(x_{i},y_{i},z_{i}\right)$, captured by the mobile tag at $\left({\hat{x}}_{p},{\hat{y}}_{p},{\hat{z}}_{p}\right)$, and that the signal propagates in a straight line, acquiring the set of straight line paths $\left\{l_{1},l_{2},\cdots ,l_{i}\right\}$ between the mobile tag and the ranging base stations;
(4) Obtain the set $\left\{N_{1},N_{2},\cdots ,N_{i}\right\}$ of geometries in the environment that intersects with all propagation lines; if the set is non-empty, proceed to step (5); otherwise, proceed to step (6);
(5) Acquire all the relevant parameters related to UWB signal penetration for the geometries in set $N_{i}$ and perform environmental compensation correction on the measured distance $d_{i}$, updating the measured distance set $\left\{d_{1}^{n},d_{2}^{n},\cdots ,d_{i}^{n}\right\}$;
(6) Apply distance filtering to the corrected distance set $\left\{d_{1}^{n},d_{2}^{n},\cdots ,d_{i}^{n}\right\}$ to obtain the set of distances $\left\{d_{1},d_{2},\cdots ,d_{i}\right\}$ used for spatial position calculation;
(7) Based on the coordinates $\left(x_{i},y_{i},z_{i}\right)$ of the base station tags and the distance set $\left\{d_{1},d_{2},\cdots ,d_{i}\right\}$, use the trilateration algorithm to obtain the spatial position calculation result $\left(x_{p}^{n},y_{p}^{n},z_{p}^{n}\right)$, and update the estimated spatial position $\left({\hat{x}}_{p},{\hat{y}}_{p},{\hat{z}}_{p}\right)$;
(8) If the difference between two consecutive estimated spatial positions is less than a threshold, proceed to step (9); if the position difference is greater than the threshold, but the number of iterations is less than the threshold, proceed to step (3); otherwise, positioning fails, proceed to step (10);
(9) We use the final positioning results to update the spatial position $\left(x,y,z\right)$ of the mobile tag and use consecutive positioning results to update the velocity ${\left[\begin{array}{ccc} v_{x} & v_{y} & v_{z} \end{array}\right]}^{T}$ of the mobile tag and use it as the initial state for the next IMU calculation;
(10) Proceed with the next positioning cycle.

## 3. Experiments and Results

### 3.1. Experimental Equipment and Environment

**Figure 9:**
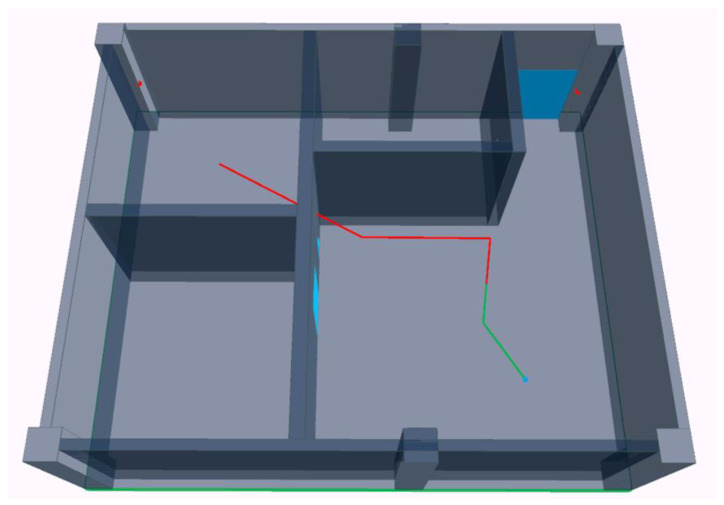
Experimental environment. Schematic diagram of the experimental environment, the green trace segment indicates that the tag communicates with the base station in an LOS environment, and the red trace segment indicates that the tag communicates with the base station in NLOS conditions.

To validate the integrated positioning approach, an empirical experiment was carried out. The main controller of the positioning device was the STM32F103, and the UWB hardware was the DecaWave DW1000, which achieved a ranging accuracy of ±5 cm. The IMU hardware, ICM-42605, was capable of outputting three-axis acceleration and angular velocity data with a gyroscope noise level of 0.0038 dps/rt-Hz and an accelerometer noise of 70 µg/rt-Hz. The digital environment for the experiment was constructed using the Building Information Model of the test scenario, as presented in Figure 9. The experiment took place in a civil engineering laboratory, where the main obstacles included a 30 cm thick concrete wall and a 5 cm thick fireproof iron door. The base stations were composed of the main controller and the DW1000, while the tags included the main controller, DW1000, and ICM-42605. The DW1000’s ranging frequency was set to 1 s, and the ICM-42605’s sampling rate was 0.01 s. As illustrated in Figure 9, the experimental environment was equipped with four base station tags and one mobile station tag. The mobile station tag followed a predetermined path, where the green segments of the trajectory represented an LOS environment and the red trajectory included segments with NLOS conditions.

### 3.2. Dynamic Positioning Experiment and Results

#### 3.2.1. Distance Measurement Results

**Figure 10:**
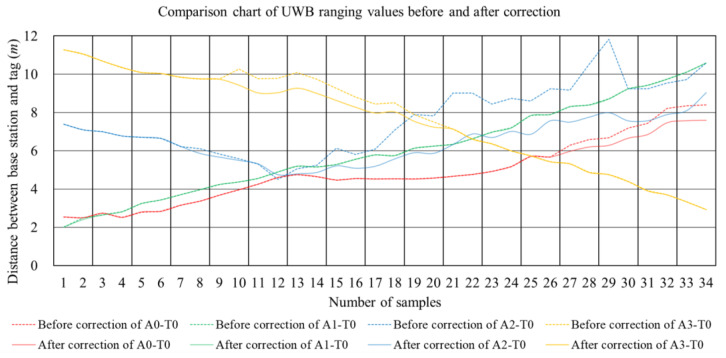
Comparison chart of UWB ranging values before and after correction.

Dynamic positioning experiments and their subsequent analysis are pivotal in understanding the efficacy of the positioning system. As shown in Figure 10, the *x*-axis represents the number of samples, and the *y*-axis represents the distance between the tag and different base stations. The dashed line represents the distance before correction, and the solid line represents the distance after correction. The base station A0 and the tag are in the NLOS environment after 25 range measurements. The base station A1 and the tag are in the LOS environment throughout the measurement process. The base station A2 and the tag are in the NLOS environment during the 8th to 13th range measurements. The base station A3 and the tag are in the NLOS environment during the 8th to 21st range measurements. This is basically consistent with the LOS environment of the positioning result location. When the tag and the base station are in the NLOS environment, the range measurement results will be significantly corrected, with smaller corrections in some cases.

#### 3.2.2. Positioning Test Results

The positioning error statistics for different positioning methods are shown in Table 1, Table 2 and Table 3. In an entirely LOS environment, the three different positioning methods demonstrate high accuracy, and their errors are not significantly different from each other. In scenarios including NLOS environments, the fusion positioning method of IMU/environment/UWB significantly outperforms the other two positioning schemes. In conclusion, the proposed fusion positioning method demonstrates superior positioning results in test scenarios, including NLOS environments. Compared to standalone UWB positioning and the IMU/UWB fusion positioning method using the ESKF algorithm, the positioning accuracy in the XY plane has increased by 295% and 267%, respectively.

**Figure 11:**
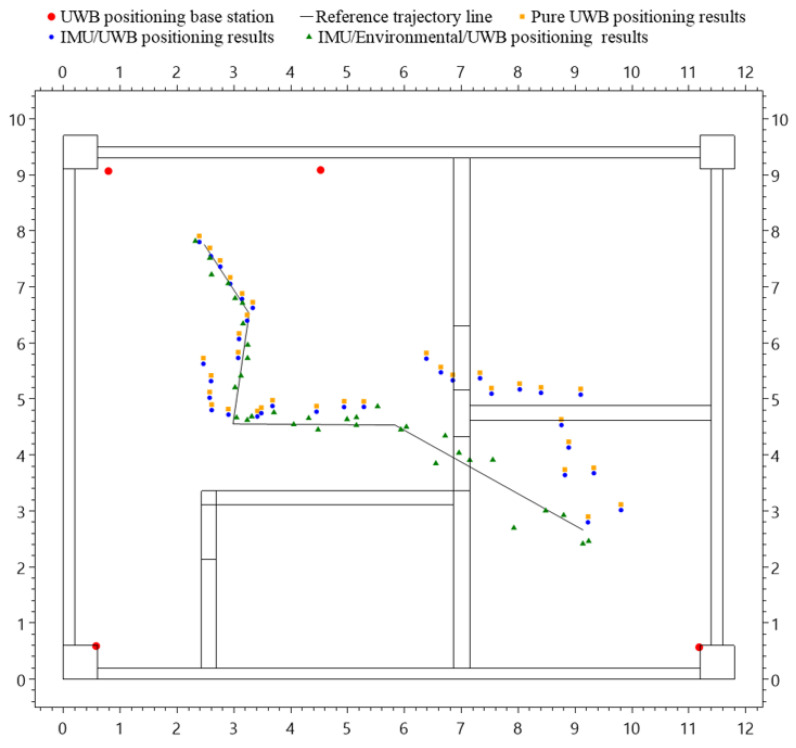
Dynamic trajectory chart calculated by different positioning methods.

For the purposes of this study, the focus is predominantly on comparing and analyzing the positioning progress on the XY plane. Figure 11 illustrates the planar trajectories determined using various methods. Specifically, the red dots signify the position of the Ultra-Wideband (UWB) positioning base stations. The solid black line delineates the reference trajectory. Orange squares depict the trajectory based solely on UWB positioning. Blue dots represent the trajectory derived from an integrated IMU/environment/UWB positioning. Green triangles indicate the trajectory from the combined IMU/environment/UWB positioning approach.

## 4. Discussion

### 4.1. Positioning Result Analysis

Due to the height limitations in the indoor environment, the small horizontal height differences between base stations significantly interfere with the *z*-axis positioning accuracy of the Triangulation (Three-sided) positioning algorithm. Consequently, the *Z*-axis coordinates derived from the positioning methodology proposed herein remain unreliable.

In an entirely LOS environment, the three different positioning methods demonstrate high accuracy, and their errors are not significantly different from each other. Among them, the IMU/UWB fusion positioning has the highest accuracy, followed by the IMU/environment/UWB fusion positioning, while the pure UWB positioning has the lowest accuracy. In scenarios that include NLOS environments, the accuracy of both pure UWB positioning and IMU/UWB fusion positioning drastically decreases, exhibiting “jump-like” deviations from the true trajectory at environmental boundaries. While the IMU/UWB fusion provided some error correction capability, its performance was not ideal. Implementing digital environmental corrections enhanced the trajectory alignment with the true path. However, signals traversing walls during movement did not encounter uniform mediums and were further impacted by non-digitized experimental environment disturbances. Although this study uses a digital environment correction model to partially correct UWB ranging results, there is still a significant difference compared to the actual distance, especially at the junctions of different environments. Furthermore, as the duration of the tag’s stay in NLOS environments increases, the positioning results gradually diverge, which could even lead to positioning failures. In conclusion, the fusion positioning method proposed in this study shows promising positioning results in test scenarios that include NLOS environments.

### 4.2. Limitations and Future Work

The fusion positioning method proposed in this paper utilizes the digital environment to provide correction information for pulse signals when penetrating structures. However, the calculation of the relationship between the propagation path and the geometric entities of structures involves a complex process. As the positioning environment expands, the number of geometric entities will also increase, leading to unnecessary computations when traversing all entities. To address this, associating base stations with spatial entities based on the effective coverage range of base station signals can reduce unnecessary computations, enhancing the efficiency of this positioning method.

The building components in the test environment of this article are all regular spatial bodies, and this method will not be applicable when correcting the distance measurement results for irregular spatial bodies. Additionally, for the correction information of pulse signals penetrating structures, this paper uses linear fitting based on multiple measurements of structures in the experimental environment. With a limited number of experimental samples, recalibration of structures in a new positioning environment is required. In future research, a more refined error model can be developed by considering the dimensions, materials, electromagnetic properties, reflection coefficients, absorption coefficients, etc., of structures through which pulse signals penetrate. This approach aims to enhance the precision of the positioning system and reduce the complexity of its setup.

## 5. Conclusions

Based on the current UWB signal propagation characteristics and research aimed at mitigating the impact of UWB-based positioning under NLOS environments, we introduced a novel positioning method. This method integrates digital environmental interference into the position calculation process. The primary goal is to provide precise compensation for ranging errors across varied scenarios. Through iterative refinement, we achieved superior positioning accuracy within NLOS environments.

Our proposed positioning approach accounts for the influences of different environmental conditions on moving tags with base station tags during the ranging process. This consideration led to enhanced positioning outcomes when tested in NLOS scenarios. However, it is pertinent to note that the environmental correction parameters we employed demand multiple measurements for determination. Different positioning environments exhibited significant variations in these correction parameters, posing challenges in the system establishment.

For future research directions, it is imperative to explore the generalized patterns of UWB signal penetration through diverse obstacles. Recording this invaluable data in a digital environmental model will enhance the accuracy of positioning signal propagation insights. Such advancements promise to simplify the complexities associated with system development, extending the broad applicability of our positioning methodology.

## Figures and Tables

**Table 1:** Pure UWB Positioning Error Statistics.

Error	LOS Environment			Including NLOS Environment		
	Maximum Value	Minimum Value	Average Value	Maximum Value	Minimum Value	Average Value
*X*-axis (m)	0.091	0.009	0.053	1.700	0.03	0.683
*Y*-axis (m)	0.270	0.141	0.199	1.531	0.007	0.704
*Z*-axis (m)	1.042	0.253	0.639	2.336	0.921	1.628
XY plane (m)	0.270	0.142	0.208	2.288	0.291	0.998

**Table 2:** ESKF Algorithm Implemented IMU/UWB Fusion Positioning Error Statistics Table.

Error	LOS Environment			Including NLOS Environment		
	Maximum Value	Minimum Value	Average Value	Maximum Value	Minimum Value	Average Value
*X*-axis (m)	0.092	0.008	0.051	1.700	0.031	0.684
*Y*-axis (m)	0.169	0.041	0.091	1.431	0.109	0.612
*Z*-axis (m)	1.023	0.003	0.501	2.335	0.914	1.627
XY plane (m)	0.169	0.044	0.108	2.222	0.22	0.933

**Table 3:** IMU/Environment/UWB Fusion Positioning Error Statistics Table.

Error	LOS Environment			Including NLOS Environment		
	Maximum Value	Minimum Value	Average Value	Maximum Value	Minimum Value	Average Value
*X*-axis (m)	0.198	0.061	0.104	0.878	0.009	0.219
*Y*-axis (m)	0.187	0.015	0.073	0.493	0.003	0.203
*Z*-axis (m)	0.620	0.048	0.404	2.177	0.007	0.703
XY plane (m)	0.221	0.065	0.134	0.976	0.071	0.319

## Data Availability

No data were used to support this study.
